# 

**Published:** 1883-06

**Authors:** 


### CONTENTS.

| | PAGE. |
|---|---|
| Climate for Consumptives | 125 |
| Sour Stomach | 126 |
| Regulating the Bowels | 127 |
| Care of the Feet | 128 |
| Measles | 129 |
| Acid Drinks | 131 |
| Snakes and Snake Poison | 132 |
| Agnosticism in China | 134 |
| Tomato Flour | 135 |
| How "Greasers" Live | 135 |
| The National Park | 136 |
| Why There are no Water-Rats in Ireland | 138 |
| Small Wa's s | 139 |
| Leave-Taking | 140 |
| Food Adulteration | 142 |
| Hair as a Means of Classifying Races | 143 |
| Trees by the Roadside | 144 |

